# The Laryngoscope

**Published:** 1860-08

**Authors:** 


### THE LARYNGOSCOPE.

(From the Berlin correspondence Medical Times and Gazette.)

As far as I am acquainted with the periodical literature of our profession, no notice has as yet appeared in your columns or in those of your cotemporaries, with regard to the highly practical results obtained on the continent by the use of the laryngoscope.

Having had occasion to convince myself of the comparative facility with which the larynx can be explored by means of this simple contrivance, I feel confident that its importance for the diagnosis of laryngeal disease cannot be overrated, and it will be a mere truism to state that we shall be able to attack effections of the larynx with far greater discrimination and success, if the uncertainties, inseparable from a symptomatic diagnosis, can thus be replaced by the precise results which a distinct view of the affected parts must afford.

A few weeks ago I was present at a post-mortem of a phthisical individual, whose larynx had been carefully examined, a short time prior to decease, by Professor Traube. The changes found in the larynx bore testimony to the accuracy of the results obtained by laryngoscopic investigation. The following remarks on the instrument and its application, are mainly extracted from a monograph, published in the early part of the year, by Professor Czermak, who, together with Dr. Turck, of Vienna, has the great merit of having re-directed the attention of the profession to this important means of diagnosis. Indeed these Viennese physicians may be said to have re-invented the larynx-speculum. Apart from its decided practical usefulness, the fact of the laryngoscope being originally an English invention ought to stimulate English surgeons to take an active part in the reform of laryngo-pathology, to which the general application of the instrument is likely to lead.

In Liston's Practical Surgery, page 417, we read, under the head of Ulcerated Glottis, the following remarks: "A view of the parts may be sometimes obtained by means of a speculum—such a glass as is used by dentists—on a long stalk, previously dipped in hot water, introduced with its reflecting surface downward, and carried well into the fauces."

This pregnant hint of Liston's remained unnoticed till 1855, when Garcia published a most valuable series of auto-laryngoscopic investigations, instituted for the purpose of elucidating the mechanism of the human voice. In these experiments the image of the larynx was reflected from a mirror placed against the soft palate, so as to be received upon a second mirror placed in front of the observer, (auto-laryngoscopy.) An elementary knowledge of cateptrics will suffice to explain the principles upon which Liston-Garcia's method of investigation is founded.

The examination itself is conducted in the following manner: A metallic mirror, varying in size from six to fourteen lines in diameter, in shape either square with rounded edges, as recommended by Czermak, or oval, according to Turck's proposal, or, as it has been found very convenient by Dr. Levin, of Berlin, semi-circular, with a concave inferior margin, soldered to a slightly flexible metallic handle, is to be introduced into the well-opened mouth, and fixed in such an angle against the uvula and soft palate as to throw incident luminous rays upon the larynx, and to reflect an image of the parts thus illuminated into the eye of the observer. To prevent the mirror from becoming dim by condensation of vapor upon its surface, it is necessary to warm it previous to introduction, by dipping it into hot water, or holding the unpolished surface over the flame of a small spirit lamp. Garcia made use of the direct rays of the sun in his experiments; as this source of illumination, however, is not always available, and, even if so, attended with obvious inconveniences in practice, Czermak proposes the use of a perforated concave mirror of 7—12 sec. focal distance, by which the light of an ordinary lamp can be concentrated upon the larynx-speculum, the eye of the observer being applied to the perforation. As the distinctness of the image will depend upon the brilliancy of the illumination employed, it will be found advantageous to concentrate the light of the lamp upon the concave mirror, by means of a powerful bi-convex lens. Dr. Levin, of this city, has devised a highly convenient apparatus for this purpose, consisting of a tin tube carrying a convex lens of two and a half inches focal distance, and about the same diameter, which, by means of a simple contrivance, can be fixed horizontally over an Argand lamp, after the shade has been removed.

The perforated concave reflector can either be held between the teeth of the observer, fixed on a suitable ivory handle, as recommended by Czermak, or attached to a large spectacle frame, according to Stellwag's proposal, or it can be suspended from a support screwed to the corner of the table on which the lamp is placed. The latter contrivance will be found the most convenient for practical purposes. I think it was first introduced by Dr. Levin.*

It will be most convenient to place the lamp to the right of the patient, who is to be examined in the sitting posture, his hands resting upon his knees, his body slightly advanced and his head slightly reclining backward. According to Professor Traube's advice, the lamp, concave mirror, and larynx-speculum ought to be on the same level, and the angle formed by the rays incident upon, and reflected from, the concave mirror as acute as possible. On this account it will be wise to place the lamp a little behind the patient. The observer supports the head and chin of the patient with his left, and introduces the larynx speculum with his right hand, looking through the perforation of the concave mirror, by means of which he illuminates the pharynx.

By causing the patient to sound alternately the Roman vowels, *a. e.*, the velum and uvula will be raised so as to admit of the mirror being introduced with greater facility. In pressing the speculum against the soft palate and uvula, great care must be taken to avoid touching the posterior wall of the pharynx, the palatine arches, and the base of the tongue, to prevent the supervention of vomiting and deglutition. "In this manner," as Czermac says, "it is possible to look into the very depths of the pharynx, to obtain a distinct image of the individual parts of the larynx, and, as I first demonstrated in my own person, to see the bifurcation of the trachea reflected through the widely opened glottis, with the tracheal rings shining through the thin mucous membrane."

Of course, considerable practice and a certain amount of dexterity are required for successful handling of the laryngoscope, notwithstanding the simplicity of the principle, upon which the method is founded.

The difficulties are mainly owing to the great irritability of the palate, which, in some individuals, is so considerable as not to tolerate the contact of a foreign body; others are unable to keep their mouths open for any length of time, or to command the position of the tongue, which ought to be well flattened and protruded. Some patients, as Professor Traube correctly remarks, suffer from a kind of "moral nausea," threatening to vomit as soon as they are told to open their mouths. This extreme irritability can be overcome by methodically accus-

---

* Mr. Yearsley has requested us to state that he has used Mr. Avery's ear lamp in this way, for several years past.—Ed.

toming the parts to the contact of foreign bodies, as it is often requisite prior to surgical operations on the palate. I remember reading that bromide of potash has the power of lowering the sensibility of the pharyngeal mucous membrane; it might deserve a trial in very refractory cases.

In general, however, the irritability of uvula and soft palate will be found very inconsiderable, so that they can be raised and pressed against the posterior wall of the pharynx without any inconvenience to the individual experimented upon. In Professor Traube's clinic I have seen an individual sitting for nearly ten minutes with the larynx-speculum applied to the fauces, so that fifteen medical men who were present could successively examine the reflected image of the glottis without any reflex phenomena supervening to interrupt the observations.

In this case the mouth of the patient was held open by a very convenient instrument, devised by Dr. Levin. The handle of the larynx mirror is attached by a ball-hinge to the upper bar of the mouth speculum, so as to admit of the larynx mirror being easily adjusted for the purpose of demonstration.

In the fifth chapter, Czermak details his method for obtaining a view of the posterior surface of the velum, the naso-pharyngeal cavity, etc., and he represents the image obtainable by rhinoscopic investigation, the commencement of the Eustachian tubes being also rendered visible. Wilde has already investigated the latter by a similar method.

To obtain an image of these parts, a speculum must be introduced under the velum, with its reflecting surface turned obliquely upwards, so as to illuminate the naso-pharyngeal cavity. A speculum is proposed for this purpose, to which a sliding wire-hook is attached for the purpose of raising the velum.

Examinations of this kind are, of course, surrounded by numerous difficulties, and can only be expected to succeed if a combination of favorable circumstances obtains.

The auto-laryngoscopic observations instituted by Czermak for physiological purposes, are mainly confirmative of the results obtained by Garcia's celebrated investigations, and his work will amply repay perusal to those who are interested in the important questions involved in the study of the mechanism of the human voice.

The pathological observations which conclude the work, twenty in number, illustrating most varied and interesting forms of laryngeal disease, as revealed by the larynx speculum, are calculated to convince the most sceptical of the great advantages which must accrue to the practitioner from the adoption of this method of investigation.

The possibility of the eye serving as a guide for the hand in the topical treatment of affections of the larynx and deep parts of the pharynx, is also proved by some of these observations. You must permit me to reserve my detailed statement for a future communication. Two of these cases, the first and third, during the course of which laryngotomy had to be performed, on account of steonsis of the larynx, are of particular interest, being the first in which, by a novel adaptation of laryngoscopy, the glottis was investigated from below. This was affected by introducing a small mirror attached to a suitably bent handle, with its reflecting surface turned upward, into a fenestrated tracheotomy tube. By illuminating this speculum with a concave reflector, the most brilliant and accurate images of the lower aspect of the glottis, etc., were obtained, and the nature of the pathological changes affecting the parts clearly ascertained. This method promises to be of great importance for the diagnosis and treatment of deep-seated affections of the larynx, particularly in cases of laryngeal tumors which cannot be attacked from above. By reversing the reflecting surface of the mirror introduced into the tracheotomy tube, the deep parts of the trachea might also be explored.

